# Anisotropic Effects on the Thermoelectric Properties of Highly Oriented Electrodeposited Bi_2_Te_3_ Films

**DOI:** 10.1038/srep19129

**Published:** 2016-01-18

**Authors:** Cristina V. Manzano, Begoña Abad, Miguel Muñoz Rojo, Yee Rui Koh, Stephen L. Hodson, Antonio M. Lopez Martinez, Xianfan Xu, Ali Shakouri, Timothy D. Sands, Theodorian Borca-Tasciuc, Marisol Martin-Gonzalez

**Affiliations:** 1 IMM – Instituto de Microelectrónica de Madrid (CNM-CSIC), Isaac Newton 8, PTM, E-28760 Tres Cantos, Madrid, Spain; 2 Birck Nanotechnology Center, Purdue University, West Lafayette, IN, United States; 3 School of Mechanical Engineering, Purdue University, West Lafayette, IN, United States; 4 Departament d’Enginyeria Electrònica, Universitat Politècnica de Catalunya, EPSEVG, 08800 Vilanova i la Geltrú, Spain; 5 School of Electrical and Computer Engineering, Purdue University, West Lafayette, IN, United States; 6 Mechanical, Aerospace and Nuclear Engineering Department, Rensselaer Polytechnique Institute, Troy, New York 12180, USA

## Abstract

Highly oriented [1 1 0] Bi_2_Te_3_ films were obtained by pulsed electrodeposition. The structure, composition, and morphology of these films were characterized. The thermoelectric figure of merit (zT), both parallel and perpendicular to the substrate surface, were determined by measuring the Seebeck coefficient, electrical conductivity, and thermal conductivity in each direction. At 300 K, the in-plane and out-of-plane figure of merits of these Bi_2_Te_3_ films were (5.6 ± 1.2)·10^−2^ and (10.4 ± 2.6)·10^−2^, respectively.

Thermoelectric materials have garnered significant attention in response to the necessity to develop alternative energy sources than those that rely on fossil fuels. The performance of thermoelectric materials^1^ is quantified by their figure of merit, which is determined by the Seebeck coefficient (*S)*, electrical conductivity (*σ)*, and thermal conductivity (*κ)*. The figure of merit is formulated as 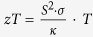, where *T* is the absolute temperature.

Bi_2_Te_3_-based alloys are thermoelectric materials well suited for applications that operate in the temperature range between room temperature and 100 °C^2^. Bi_2_Te_3_ is a semiconductor with a band gap of 0.15 eV^3^ and a rhombohedral-hexagonal structure. The unit cell parameters of this semiconductor at room temperature are a = 3.8 Å and c = 30.5 Å. Five layers are observed along the *c-axis*: Te(1)-Bi-Te(2)-Bi-Te(1) (see Fig. 1(A)). The bonds between Te and Bi atoms are strongly covalent while adjacent Te atoms are bonded by van der Waals interactions. The crystallographic orientations of the films featured in this work are shown on Fig. 1(B).

In this crystal structure, the electrical^4^ and thermal conductivities^5^ are highly anisotropic^6^, while the Seebeck coefficient is nearly isotropic^7^. The electrical and thermal conductivities in the direction perpendicular to the substrate are higher than the thermal and electrical conductivities in the direction parallel to the substrate. These directional effects that dictate the anisotropic properties of Bi_2_Te_3_ are commonly observed in the research community. In the work of Antonova *et al*.^8^, the room temperature anisotropic thermoelectric properties of single-crystalline bulk bismuth telluride oriented parallel and perpendicular to the *c-axis* were highlighted. The figure of merit parallel to the *c-axis* was determined to be 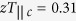, with Seebeck coefficient, electrical conductivity, and thermal conductivity values of −240 μV/K, 0.02 (μΩ·m)^−1^, and 1 W/m·K^8^, respectively. Conversely, when the crystal structure was oriented perpendicular to the *c-axis*, the values of the Seebeck coefficient, electrical conductivity, and thermal conductivity were −240 μV/K, 0.1 (μΩ·m)^−1^, and 2.2 W/m·K, respectively, and corresponded to a figure of merit of 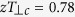
8. The increase in the figure of merit by a factor of 2.5 from the parallel to perpendicular orientations is primarily due to a significantly larger increase in the electrical conductivity relative to the increase in thermal conductivity. Fleurial *et al*.^9^ experimentally obtained an approximately fourfold increase in the electrical conductivity from the parallel to perpendicular directions, which corresponds well with theoretical values published by Goldsmith *et al*.^5^ and Jacquot *et al*.^10^. The increase in thermal conductivity is typically lower than that for the electrical counterpart, with a handful of publications reporting a factor 2^5^,^8^,^9^ or 3^10^ increase from the parallel to perpendicular orientations. Thus, the anisotropic properties of Bi_2_Te_3_ films can be exploited to achieve maximum performance.

In this work, the thermoelectric properties of electrodeposited Bi_2_Te_3_ films were measured in the parallel and perpendicular orientations relative to the substrate. The film was confirmed to be highly oriented along the [1 1 0] direction by X-ray diffraction. Electrodeposition was chosen as the preferred fabrication method due to its capability to inexpensively yield thermoelectric materials at room temperature, which can potentially be advantageous for scalable manufacturing. The figure of merit was fully determined in both directions and the anisotropy of the electrical and thermal conductivities as well as the isotropy of the Seebeck coefficient were investigated experimentally. Additionally, comparisons to experimental and theoretical results obtained for single crystals are included in this work.

## Results and Discussion

**Figure 2:**
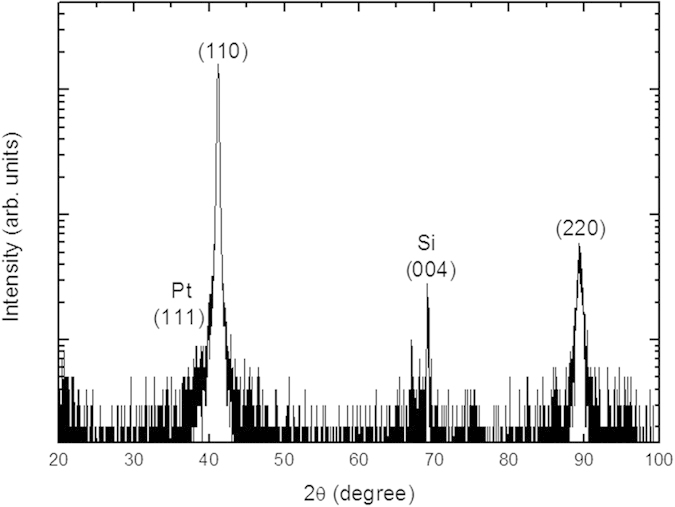
X-ray diffractograms of Bi_2_Te_3_ film grown at pulsed electrodeposition. The diffractogram y-axis is in log scale to hihglight that no other orientation is presented in the film.

The fabrication process was performed with pulsed electrodeposition at a constant potential over 0.1 s and zero current density over 0.01 s. The applied potential was + 0.05 V relative to Ag/AgCl, which matches the conditions used in the work of Manzano *et al*.^11^. Figure 2 shows a logarithmically scaled X-ray diffractogram to enhance the diffraction peaks of lower intensity and verify that minor diffraction peaks due to impurities or other orientations are comparably negligible. The diffraction peak at 2*θ* = 69.12° corresponds to the Si (JCPDS 27–1402) substrate. The two diffraction peaks at 2*θ* = 41.17° and 89.36° are attributed to the 110 and 220 orientation planes, respectively, and indicate that the film is highly oriented along the [1 1 0] direction.

**Figure 3:**
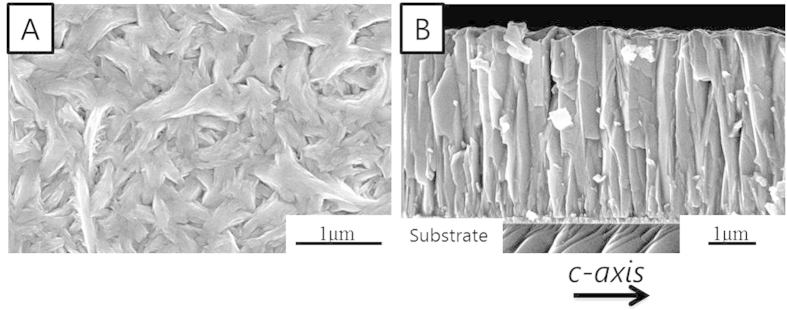
SEM images of Bi_2_Te_3_ film. (**A**) top view, and (**B**) cross section.

In the scanning electron microscopic images (Fig. 3(A,B)), top and cross-sectional views of the Bi_2_Te_3_ film are shown, respectively, and indicate that the morphology of the film is columnar with particle sizes on the order of microns. The thickness measured from the cross-section images is approximately 4 μm. To accurately determine the electrical and thermal conductivities, the thickness of the Bi_2_Te_3_ film should be quantified with high fidelity. Therefore, the thickness of the Bi_2_Te_3_ film was also measured using a profilometer, which yielded a thickness value of 4.5 μm ± 0.3 μm. The roughness of the film was approximately 300 nm, which is smoother than films typically grown by electrodeposition. The smoother finish can be attributed to the pulsed electrodeposition at constant potential and zero current density employed in the growth process. The chemical composition of the film was analyzed by EDX at two separate areas of the film. The atomic percentages of bismuth and tellurium were 40 ± 2% and 60 ± 2%, respectively, which is expected for ideal thermoelectric properties of Bi_2_Te_3_.

### In-plane Power Factor: Measured Parallel to the Substrate Plane

The in-plane electrical resistivity at room temperature was measured to be 15.0 ± 1.5 μΩ·m, indicating that the electrical conductivity is on the order of (6.7 ± 0.7) ·10^−2^ (μΩ·m)^−1^. The value of the electrical conductivity is a factor of two higher than the value obtained by Jacquot *et al*.^10^ for calculated transport properties of a single crystalline structure. The Seebeck coefficient was measured as -58 ± 4 μV/K, which is indicative of n-type behavior. While the Seebeck coefficient is lower than that of a single crystalline structure^10^, the value is of the same order of bismuth telluride films grown by electrodeposition without a complexing agent^11^. The power factor at room temperature, which is defined as S^2^·σ, was determined to be 225 ± 32 μW/m·K^2^ and is lower than values reported in the literature for films grown under similar conditions^12^,^13^. This discrepancy is likely due to a less electrically conducting film^11^,^12^.

**Figure 4:**
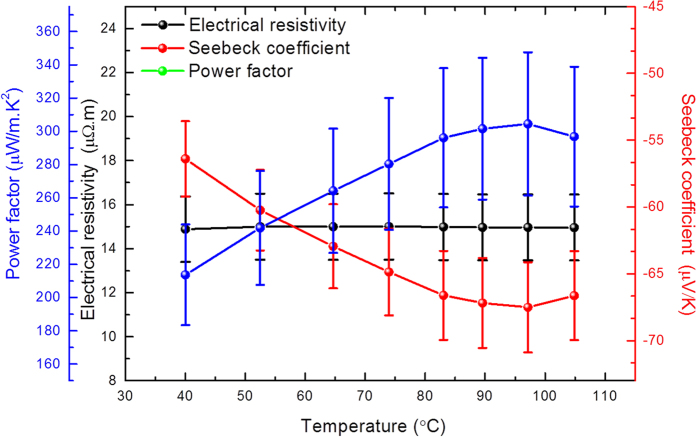
Electrical resistivity, Seebeck coefficient, and Power factor measured in-plane as a function of temperature.

The in-plane electrical resistivity and Seebeck coefficient were measured within a temperature range of 37 °C to 107 °C using a commercial LSR-3 Linseis^®^ system. In Fig. 4., the electrical resistivity is displayed on the primary axis while the Seebeck coefficient is displayed on the secondary axis. The electrical resistivity is constant within this temperature range with a value of 15.0 ± 1.5 μΩ·m, while the Seebeck coefficient becomes increasingly negative as the temperature increases, reaching a maximum value of -67.0 ± 3.4 μV/K at 97 °C.

As determined from the in-plane electrical resistivity and Seebeck coefficient measurements, the in-plane power factor increases with temperature as shown in Fig. 4, also reaching a maximum value of 300 ± 42 μW/m·K^2^ at 97 °C. The values of the thermoelectric properties measured in-plane are the result of the measurement along the [0 0 1] direction randomly distributed in x–y plane. It must also be taken into account that in-plane measurements in these films will be influenced by the presence of grain boundaries. So here we should not consider only [0 0 1] orientation, such as for a single crystal.

The in-plane mobility and carrier concentration of the film were determined by Hall effect measurements, using van der Pauw configuration. The values obtained and the single crystal bulk values found in literature are shown in the Table 1.

The in-plane mobility and carrier concentration of the Bi_2_Te_3_ film obtained by electrodeposition are of the same order of magnitude for bismuth telluride single crystal bulk materials, which is interesting when considering that these electrodeposited films present grain boundaries which may affect the electrical conductivity.

### Out-of-plane Power Factor: Measured Perpendicular to the Substrate Plane

The out-of-plane electrical resistivity and Seebeck coefficient were also measured at room temperature. The out-of-plane electrical conductivity is (3.2 ± 0.4)·10^−1^ (μΩ·m)^−1^^13^, which is approximately a factor five (4.8) higher than the in-plane electrical conductivity. An in detail description on this measurement and the COMSOL® simulation to extract this value can be found on reference 13. The fivefold increase between the out-of-plane and in-plane electrical conductivities is comparable to those obtained by Goldsmid *et al*.^5^ and Fleurial *et al*.^9^. The fivefold increase is also approximately 1.3 times larger than the increase reported by Jacquot *et al*.^10^.

**Figure 5:**
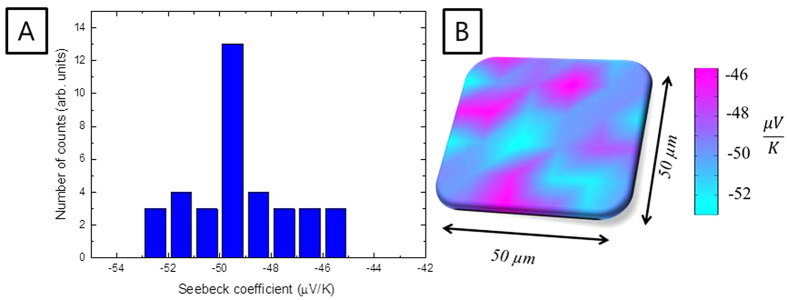
Seebeck coefficient out-of-plane at room temperature. (**A**) Seebeck coefficient distribution, and (**B**) Seebeck coefficient map as measured by the commertial system Seebeck microprobe.

The mean value of the out-of-plane Seebeck coefficient was measured to be approximately -50 ± 5 μV/K as can be observed in the histogram in Fig. 5. This value is within the experimental error of the value obtained for the in-plane direction at room temperature, confirming the isotropic behavior of the Seebeck coefficient of electrodeposited Bi_2_Te_3_ films. This isotropic behavior is in consonance with the values obtained in single crystal bulk Bi_2_Te_3_^10^.

The out-of-plane power factor was 800 ± 189 μW/m·K^2^ at room temperature. This is the highest value obtained in Bi_2_Te_3_ films grown by electrodeposition, which is primarily attributable to a dramatic increase in the electrical conductivity since the Seebeck coefficient is nearly isotropic.

### Thermal Conductivity Along the [1 1 0] and [0 0 1] Directions

The thermal conductivity, which is a difficult thermoelectric property to measure in thin films, was obtained in both directions using two different techniques: (i) time domain thermoreflectance (TDTR) and (ii) photoacoustic (PA). The TDTR method rendered an out-of-plane thermal conductivity of 2.4 ± 0.2 W/m·K while the PA method yielded an out-of-plane thermal conductivity of 2.3 ± 0.2 W/m·K. In order to ensure a highly reflective surface for the TDTR measurements, the films were detached from the substrate^14^,^15^ as this sample face presents a surface flatness of around 10–20 nm. According to the literature^16^ and taking into account the frequencies used to perform the TDTR measurements on the films (0.824 MHz and 6.58 MHz), the heat penetration depth range should be from 200 nm–700 nm for the Bi_2_Te_3_ films. As the thickness of the sample is much higher than the heat penetration depth, 4 μm, no substrate contribution influences the thermal conductivity measurements. The values obtained by both techniques are similar and comparable to values in the literature for single crystal bulk Bi_2_Te_3_^7^,^10^. In order to measure the thermal conductivity along the [0 0 1] direction, a bismuth telluride film preferentially oriented along the [0 0 1] direction was prepared by changing the growth time in the electrodeposition cycle with zero current density. The film was tailored to have the same thickness and characteristics as the [1 1 0] film. Similarly as for the [1 1 0] oriented film, the deposition was performed by pulsed electrodeposition between reduction potential and zero current density. The crystallographic structure was controlled by changing off-time, which means the time of zero current density. In the case of the film oriented along the [1 1 0] direction, the reduction potential was applied over 0.1 s, followed by a zero current density over 0.01 s. However, in the case of the film oriented along the [0 0 1] direction, the reduction potential was applied over 0.1 s, followed by a zero current density over 0.2 s. In both cases the total time was set at 3600 s. The in-plane thermal conductivity of this film was measured by the TDTR method and rendered a value of 1.2 ± 0.2 W/m·K. The ratio between the out-of-plane and in-plane thermal conductivities is approximately 2, which is similar to the values obtained in the work of Goldsmid *et al*.^5^ and Fleurial *et al*.^9^ for single crystal bulk Bi_2_Te_3_.

### Figure of Merit

With the measured out-of-plane Seebeck coefficient, electrical conductivity, and thermal conductivity, the out-of-plane figure of merit is 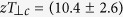 10^−2^ at 300 K, which is one order of magnitude lower than the figure of merit of single crystal bulk Bi_2_Te_3_. This discrepancy is primarily because the Seebeck coefficient of the electrodeposited films is four times lower than single crystal bulk Bi_2_Te_3_. Using the measured in-plane Seebeck coefficient, electrical conductivity, and thermal conductivity values of –58.0 ± 3.0 μV/K, (6.7 ± 0.7)·10^−2^ (μΩ·m)^−1^, 1.2 ± 0.2 W/m·K, respectively, the in-plane figure of merit is *zT*_*// c*_ = (5.6 ± 1.2)·10^−2^ at 300 K. The ratio between the out-of-plane and in-plane figures of merit is approximately 1.8, which is the same order of magnitude for single crystal bulk Bi_2_Te_3_.

## Conclusions

Bi_2_Te_3_ films highly oriented along the [1 1 0] direction were obtained by pulsed electrodeposition by changing the duration of the zero current portion of the growth cycle. At 300 K, an approximation of the in-plane figure of merit was determined to be (5.6 ± 1.2)·10^−2^ while the out-of-plane figure of merit was (10.4 ± 2.6)·10^−2^. From these measurements, the anisotropy of the electrical and thermal conductivities as well as the isotropy of the Seebeck coefficient for Bi_2_Te_3_ electrodeposited films was revealed. The electrical conductivity perpendicular to the *c-axis* is nearly five (4.8) times higher than the electrical conductivity parallel the *c-axis*. The Seebeck coefficient perpendicular to the c-*axis* is within the experimental uncertainty of the Seebeck coefficient along the c-*axis*, indicating the electrodeposited film is isotropic for this property. A twofold increase from the in-plane to out-of-plane thermal conductivities was observed. From the measured in-plane and out-of-plane values at 300 K, figure of merits of *zT*_*// c*_ = (5.6 ± 1.2)·10^−2^ and 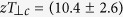
·10^−2^ are respectively rendered, which yields an increase by a factor of 1.8 between the in-plane and out-of-plane thermoelectric performances.

## Methods

### Fabrication Method

Bi_2_Te_3_ films were grown by pulsed electrodeposition in an aqueous solution using a conventional three-electrode cell. Pt wire, Ag/AgCl electrode and 150 nm Pt (1 1 1)/5 nm Cr/Si (1 0 0) were used as counter, reference and working electrode, respectively. The gold and chromium were deposited on the silicon by electron-beam evaporation. The aqueous solution used was 0.75·10^−2^ M Bi^3+^, 1·10^−2^ M HTeO^2+^ and 1M HNO_3_^17^. The solution was prepared from Sigma Aldrich^®^ Bismuth pieces (99.999%), Alfa Aesar^®^ (99.99%) Tellurium powder, and Panreac^®^ 65% nitric acid. The electrodeposition process was performed using a potentiostat-galvanostat (Eco Chemie, Model AUT302.0). In order to obtain films oriented along the [1 1 0] direction, a study focused on the pulse conditions was performed in a similar manner to Manzano *et al*.^11^. The electrodeposition was performed at a constant potential over on-time and zero current density over off-time. The total on-time was 1 hour and the experiments were carried out at 25 °C.

### Structural and Morphological Characterization

The structural properties of the films were obtained by high resolution X-ray diffraction (XRD). The measurements were performed in a Philips X´Pert four-circle diffractometer system with CuK_α_ radiation. The surface morphology and the cross-sectional structure were studied using a Philips XL305-FEG field emission scanning electron microscope (FE-SEM) with 10 KV accelerating voltage. The thickness of the films were measured by a Vecco^®^ Dektak stylus profiler system. The chemical composition was analyzed by S-3000N energy dispersive X-ray (EDX) with 20 KV accelerating voltage.

### Thermoelectric Characterization

The electrical conductivity and Seebeck coefficient parallel to the substrate plane were measured both at room temperature using an in-house system and as a function of temperature using a commercial LSR-3 Linseis system (see in the supporting information). In order to perform these measurements, the film was transferred to a glass slide using a high-temperature epoxy adhesive^14^,^15^. For the in-house system^18^,^19^, the electrical resistivity was obtained by the van der Pauw method, whereas the Seebeck coefficient was measured by applying different temperature gradients between 1 °C to 5 °C across the sample. By subtracting the slope between the generated Seebeck voltage against the temperature gradient, the Seebeck coefficient was obtained. For the commercial system, the electrical resistivity was measured by the four-probe method and the Seebeck coefficient was obtained by establishing two different temperature gradients across the sample with boundary temperatues of approximately 1 °C and 8 °C. These properties were obtained within a temperature range between ~37 °C to 107 °C in order to remain below the maximum temperature of the epoxy. Multiple measurements were performed at each temperature point in order to ensure the stabilization of the temperature gradient at the set temperature as well as improve the accuracy of the experiment.

**Figure 6:**
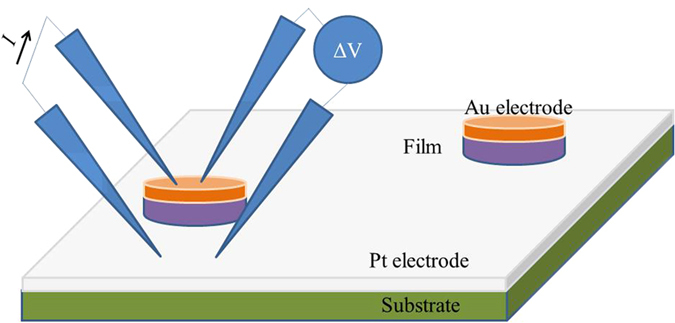
Scheme of electrical conductivity set-up for the out-of-plane direction.

The Seebeck coefficient, electrical conductivity, and thermal conductivity in the out-of-plane direction were also measured. The out-of-plane electrical conductivity of the film was measured using a four probe station. These measurements must be performed while accounting for the contact resistance and the possibility of non-uniform spreading of current across the film and electrodes. For that purpose, a custom set up was built based on a lithography process and mesa attacks to obtain disc shaped structures that help mitigate current flow in the different directions. The four point probe method measures the electrical resistivity perpendicular to the substrate plane by placing two probes on top of the disk while the other two probes are close to the mesa and on the conducting substrate. By applying a current across it with two of the probes, while measuring the voltage drop with the other ones and using a three-dimensional finite element model, which includes spreading effects along the electrodes and film structure and that takes into account the interface contact resistance, the electrical conductivity of the film perpendicular to the substrate plane could be accurately determined^13^ (see Fig. 6). This work, with an in-depth explanation, can be found in ref. 13.

Both the in and out-of-plane electrical resistance measurements were performed in DC mode, but under low applied currents (<1mA) in order to avoid the influence of the Seebeck voltage.

The Seebeck coefficient of the films were measured using a commercial Seebeck microprobe system^20^ (see in the supporting information). This technique has a heated micro-probe with a thin T-type (copper-constantan (Cu-CuNi)) thermocouple at its end and a heat sink, with another T-type thermocouple where the film is placed. When the probe is in contact with the surface of the film, a temperature gradient is generated across the thermoelectric film. The temperatures at the boundaries of the film are measured as well as the voltage drop between top and bottom thermocouple wires, i.e. Cu-Cu and CuNi-CuNi wires, respectively.

An equation for the Seebeck coefficient of the sample can be extracted from these parameters^20^,


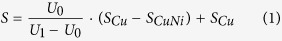


where 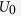
and 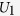
are the voltages measured from the copper (Cu) and constantan (CuNi) wires, respectively, and 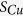
and 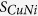 are the copper and constantan Seebeck coefficients, respectively. By translating the probe along the surface of the film, a Seebeck coefficient map can be obtained. This method was used to measure the Seebeck coefficient in the perpendicular direction to the substrate plane. However, we note that the results might be slightly influenced by the transport properties of the sample in each direction. Nevertheless, this method has been successfully applied to measure similar Bi_2_Te_3_ films^21^ and it is suitable for the current films in order to estimate the Seebeck coefficient along perpendicular direction to the substrate plane. Moreover, this technique is appropriate because the Seebeck coefficient is expected to be isotropic.

The thermal conductivity at room temperature perpendicular to the substrate plane was obtained using two different methods in order to cross-validate the results: (i) photoacoustic (PA)^23^,^24^,^25^ and (ii) time domain thermoreflectance (TDTR)^23^,^24^,^25^ in two films, one of them oriented along the [1 1 0] and the other along the [0 0 1] directions (see in the supporting information). In the photoacoustic method, incident radiation from a modulated fiber-couple laser of 980 nm wavelength with an optical power of 260 mW periodically heats the film. A layer of 80 nm of Ti was deposited by electron-beam evaporation above the film in order to absorb the radiation. The air in contact with the surface of the film expands and contracts in response to the periodic heating and acts as a thermal piston that generates acoustic waves. The acoustic waves are detected by a microphone and compared to the incident modulated signal by a lock-in amplifier. From the phase shift between both signals, the thermal properties can be delineated by applying the multilayer model developed by Hu *et al*.^26^. This technique has previously been used to measure the thermal conductivity of similar electrodeposited films^27^. In the TDTR measurements a titanium sapphire laser was employed to create pulsed laser beams with a ~12.5 ns repetition rate^20^. The laser pulses are split into pump and probe beams. The pump beam is modulated at a frequency between 0.8 and 10 MHz and creates a temperature rise on the surface of the sample. The radius of the laser beam is approximately 5.5–6.0 μm at the surface of the sample. A 70 nm aluminum transducer was coated on the sample to absorb the incident beam. The probe laser beam is the lower power laser pulse. The reflected probe beam signal was collected by a Si-photodetector and RF lock in amplifier. The obtained probe beam signal was fitted with a 3D thermal diffusion model based on thermal quadrupoles^28^. The contact resistance between Al transducer and sample surface was accounted for in the model. The laser power used during the TDTR measurements was carefully selected to create a 10 K temperature rise on the surface. We assume that the thermal properties (e.g. specific heat capacity) of the samples are consistent with the thermal properties at room temperature.

The experimental errors associated with the thermoelectric properties depend on each measurement technique. For both the in-house and the commercial LSR-3^®^ Linseis systems, a measurement error of 10% is associated with the electrical conductivity while the Seebeck coefficient has an error of 5%. The measurements of the electrical conductivity and Seebeck coefficient perpendicular to the substrate plane typically have a measurement error of approximately 10%. The thermal conductivity measured by the PA technique possesses an error of approximately 10%, which results from the assumed properties of the film (e.g. density and heat capacity), the thickness of the film, and the random error obtained by performing multiple measurements of on the film. The thermal conductivity measured by the TDTR method has an error of approximately 5–10%. Finally, both the power factor and the figure of merit errors are calculated by the propagation of these uncertainties.

## Additional Information

**How to cite this article**: Manzano, C. V. *et al*. Anisotropic Effects on the Thermoelectric Properties of Highly Oriented Electrodeposited Bi_2_Te_3_ Films. *Sci. Rep*. **6**, 19129; doi: 10.1038/srep19129 (2016).

## Supplementary Material

Supplementary Information

## Figures and Tables

**Figure 1:**
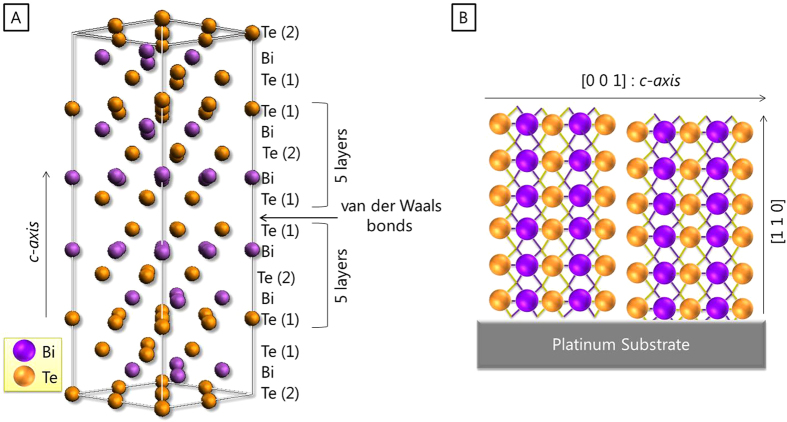
(**A**) Crystal structure of bismuth telluride. (**B**) Orientation of the electrodeposited film measured in this work.

**Table 1:** In-plane mobility and carrier concentration of Bi_2_Te_3_ film.

	This work	Single crystal bulk^9^,^29^
In-plane mobility (cm^2^V^−1^s^−1^)	29.8	31–227
Carrier concentration(cm^−3^)	3.2·10^20^	(0.07–1.5) ·10^20^

## References

[b1] Martín-González M. . Nanoengineering thermoelectrics for 21st century: Energy harvesting and other trends in the field. Renewable and Sustainable Energy Reviews 24, 288–305 (2013).

[b2] Snyder G. J. . Complex thermoelectric materials. Nat Mater 7, 105–114 (2008). 18219332 10.1038/nmat2090

[b3] Greenaway D. L. . Band structure of bismuth telluride, bismuth selenide and their respective alloys. Journal of Physics and Chemistry of Solids 26, 1585–1604 (1965).

[b4] Delves R. T. . Anisotropy of the Electrical Conductivity in Bismuth Telluride. Proceedings of the Physical Society 78, 838 (1961).

[b5] Goldsmid H. J. Recent Studies of Bismuth Telluride and Its Alloys. Journal of Applied Physics 32, 2198–2202 (1961).

[b6] Tritt T. M. . Thermoelectric Materials, Phenomena, and Applications: A Bird’s Eye View. MRS Bulletin 31, 188–198 (2006).

[b7] Rowe D. M. Materials, preparation, and characterization in thermoelectrics. (eds. Rowe D. M. .), Ch. 1, 1–4 (New York, 2012) edn.

[b8] Antonova E. E. . Finite elements for thermoelectric device analysis in ANSYS. Paper presented at 24^th^ *International Conference on Thermoelectrics*, ICT, Clemson University. Clemson, SC, USA. Publisher: IEEE.(doi: 10.1109/ICT.2005.1519922).(2005, June).

[b9] Fleurial J. P. . Thermal properties of high quality single crystals of bismuth telluride—Part I: Experimental characterization. Journal of Physics and Chemistry of Solids 49, 1237–1247 (1988).

[b10] Jacquot A. . Thermoelectric Properties as a Function of Electronic Band Structure and Microstructure of Textured Materials. Journal of Electronic Materials 39, 1861–1868 (2010).

[b11] Ma Y. . Thermoelectric properties of thin films of bismuth telluride electrochemically deposited on stainless steel substrates. Electrochimica Acta 56, 4216–4223 (2011).

[b12] Diliberto S. . Influence of pulsed electrodeposition on stoichiometry and thermoelectric properties of bismuth telluride films. Physica Status Solidi (A) Applications and Materials Science 205, 2340–2344 (2008).

[b13] Muñoz Rojo M. . High electrical conductivity in out of plane direction of electrodeposited Bi_2_Te_3_ films. AIP Advances 5, 087142 (2015).

[b14] Manzano C. V. . Thermoelectric properties of Bi_2_Te_3_ films by constant and pulsed electrodeposition. Journal of Solid State Electrochemistry 17, 2071–2078 (2013).

[b15] Caballero-Calero O. . Improvement of Bismuth Telluride electrodeposited films by the addition of Sodium Lignosulfonate. Electrochimica Acta 123, 117–126 (2014).

[b16] Koh Y. K. . Frequency dependence of the thermal conductivity of semiconductor alloys. Physical Review B 76, 075207 (2007).

[b17] Martín-González M. S. . Insights into the Electrodeposition of Bi_2_Te_3_. Journal of The Electrochemical Society 149, C546–C554 (2002).

[b18] Ares J. R. . Evolution of the Seebeck coefficient during the formation and crystallization of pyrite thin films. Journal of Physics: Condensed Matter 10, 4281 (1998).

[b19] Ferrer I. J. . Hysteresis-like behaviour of the thermoelectric voltage in photovoltaic materials. Thin Solid Films 511–512, 177–181 (2006).

[b20] Vermeersch B. . Ballistic heat transport and associated frequency dependence of thermal conductivity in semiconductor alloys. Paper presented at 13^th^ InterSociety Conference on *Thermal and Thermomechanical Phenomena in Electronic Systems. Publisher: IEEE*. San Diego, CA; United States. (DOI: 10.1109/ITHERM.2012.6231462). (2012, May).

[b21] Li S. . Fabrication of Nanostructured Thermoelectric Bismuth Telluride Thick Films by Electrochemical Deposition. Chemistry of Materials 18, 3627–3633 (2006).

[b22] Vermeersch B. . Superdiffusive heat conduction in semiconductor alloys. II. Truncated L\‘evy formalism for experimental analysis. Physical Review B 91, 085203 (2015).

[b23] Cahill D. G. Analysis of heat flow in layered structures for time-domain thermoreflectance. Review of Scientific Instruments 75, 5119–5122, doi: 10.1063/1.1819431 (2004).

[b24] Dilhaire S. . Heterodyne picosecond thermoreflectance applied to nanoscale thermal metrology. Journal of Applied Physics 110, doi: 10.1063/1.3665129 (2011).

[b25] Schmidt A. J. . Pulse accumulation, radial heat conduction, and anisotropic thermal conductivity in pump-probe transient thermoreflectance. Review of Scientific Instruments 79, 114902 (2008). 19045906 10.1063/1.3006335

[b26] Hu H. . Generalized theory of the photoacoustic effect in a multilayer material. Journal of Applied Physics 86, 3953–3958 (1999).

[b27] Abad B. . Thermoelectric properties of electrodeposited tellurium films and the sodium lignosulfonate effect. Electrochimica Acta 169, 37–45 (2015).

[b28] Cahill D. G. . Nanoscale thermal transport. Journal of Applied Physics 93, 793–818 (2003).

[b29] Yoo B. Y. . Electrochemically deposited thermoelectric n-type Bi_2_Te_3_ thin films. Electrochimica Acta 50, 4371–4377 (2005).

