# Supplementary material for: Anisotropic Effects on the Thermoelectric Properties of Highly Oriented Electrodeposited Bi2Te3 Films
Source: Sci Rep. 2016 Jan 18;6:19129. doi: 10.1038/srep19129 (PMC4726026; doi:10.1038/srep19129)
Supplement: Supplementary Information [file srep19129-s1.doc]

**Supporting Information**

**Anisotropic Effects on the Thermoelectric Properties of Highly Oriented Electrodeposited Bi_2_Te_3_ Films**

*Cristina V. Manzano^1^, Begoña Abad^1^, Miguel Muñoz Rojo^1^, Yee Rui Koh^2^, Stephen L. Hodson^2,3^, Antonio M. López Martinez^4^, Xianfan Xu^2,3^, Ali. Shakouri^2^, Timothy D. Sands^2,3,5^,* *Theodorian Borca-Tasciuc^6^, Marisol Martín-González^1,*^*

^1^ IMM – Instituto de Microelectrónica de Madrid (CNM-CSIC), Isaac Newton 8, PTM, E-28760 Tres Cantos, Madrid, Spain

^2^ Birck Nanotechnology Center, Purdue University, West Lafayette, IN, United States

^3^ School of Mechanical Engineering, Purdue University, West Lafayette, IN, United States

^4^ Escola Ponitéctina superior de Eng. Electrónica. Avda. Victor Balarguer, 1, 08800 Vilanova i La Geltrú, Spain

^5^ School of Electrical and Computer Engineering, Purdue University, West Lafayette, IN, United States

^6^ Mechanical, Aerospace and Nuclear Engineering Department, Rensselaer Polytechnique Institute, Troy, New York 12180, USA

Corresponding autor:

| Dr. Marisol Martín-González  E-mail: [marisol@imm.cnm.csic.es](mailto:marisol@imm.cnm.csic.es)  Phone number: +34 91 806 0700,  Fax number: +34 91 806 0701 |  |
| --- | --- |

A commercial equipment (Linseis LSR-3®) was used to measure the electrical resistivity and Seebeck coefficient in the in-plane direction. The precision of the measurement was periodically checked by measuring a constantan standard sample supplied by the company. Figure 1 shows that the measured values are in agreement with the expected values for both magnitudes.

Figure 1. Theoretical and experimental values of the electrical conductivity and Seebeck coefficient of a constantan sample.

The out-of-plane Seebeck coefficient was measured using a commercial system by Seebeck Microprobe®. The reliability of this system was confirmed after measuring a set of well-known Seebeck coefficient calibration samples such as Bi_2_Te_3_ n- and p-type pellets, i.e. doped with Sb (~ +180 µV/K) and Se (~ -150 µV/K). The results agree with those given by the manufacturer. Moreover, this system has been successfully used to measure Bi_2_Te_3_ thick films, as can be seen in reference [1](#_ENREF_1). The Seebeck Microprobe meets the following specifications, as given in reference [2](#_ENREF_2): Positioning accuracy: 1 µm / Reproducibility (bidirectional) 3 µm. / Travel: x-direction 150 mm, y-direction 50 mm / Local resolution of S up to10 µm, depending on the sample´s thermal conductivity/ Measuring time: <4 s per local data point / Reproducibility better than 3% of the Seebeck coefficient and electrical conductivity / Seebeck accuracy better than 5%/ Local resolution: 1 µm. In order to determine the Seebeck coefficient of our film, we performed a statistical analysis on several film locations. This rendered a Seebeck coefficient map of the surface of the sample. The average obtained from these statistics resulted in the mean Seebeck coefficient of the film. Regarding the experimental uncertainty, each Seebeck coefficient measurement carries an uncertainty but also, the standard deviation obtained from the statistical study should be also considered. According to reference the presence of the Pt substrate underneath the film presents, with much higher thermal conductivity than the Bi_2_Te_3_ film under study, makes possible a proper measurement of the Seebeck coefficient of the 4 µm film with the Seebeck microprobe with negligible influence of the substrate. Moreover, the low interface contact resistance between the Pt and the Bi_2_Te_3_ film and the low Seebeck coefficient of the Pt (~ 5µV/K) avoids important effects from this substrate.

The measurements of the electrical conductivity cross plane were performed in a four probe station with 4200-SCS Parameter Analyzer-Keithley at the IMDEA Nanoscience Institute of Madrid, which has been successfully used to determine the resistance of a wide variety of samples. It is a built-in low noise ground unit and an extremely precise measuring instrument (resolution of fA). This system, under the four probe configuration described in the manuscript, was used to determine the electrical resistance of Bi_2_Te_3_ film discs with different diameters and thicknesses. The electrical resistances obtained experimentally were matched with the results obtained from a COMSOL® Multiphysics simulation that reproduced the experimental conditions and set-up with high accuracy. In this model, the physics underlying the measuring procedure were considered, such as the dispersion of the electrical field or the influence of the interface between the electrodes and film. By matching the simulated electrical resistance with the experimental results, the electrical conductivity of the film and the interface electrode-film resistivity were accurately determined. This work, as well as the fabrication process of the film discs, can be found in reference [^3^](#_ENREF_3).

The thermal conductivity was measured by a TDTR and photoacoustic system. Both systems are calibrated before each measurement. Moreover, in order to ensure the reliability of both systems the same sample was measured by both techniques gave the following values:

- - TDTR: k = 2.4 ± 0.2 W/m·K
  - Photoacoustic: k = 2.3 ± 0.2 W/m·K

Fittings of the measurements both out of plane (by the photoacoustic technique) and in plane (TDTR performed out of plane) are shown in Figure 2 (a) and (b) respectively.

Figure 2. Experimental fittings of the samples obtained by a) photoacoustic technique (out-of plane film) b) TDTR system (in plane film).

**References**

1 Li, S. *et al.* Fabrication of Nanostructured Thermoelectric Bismuth Telluride Thick Films by Electrochemical Deposition. *Chemistry of Materials* **18**, 3627-3633 (2006).

2 Platzek, D. *et al.* in *Materials Science Forum* Vol. **492** 587-592 (2005).

3 Rojo, M. M. *et al.* High electrical conductivity in out of plane direction of electrodeposited Bi_2_Te_3_ films. *AIP Advances* **5**, doi:10.1063/1.4928863 (2015).
